# Quantitative Evaluation of the Dispersion of Graphene Sheets With and Without Functional Groups Using Molecular Dynamics Simulations

**DOI:** 10.1186/s11671-016-1336-6

**Published:** 2016-03-10

**Authors:** JinHyeok Cha, Woomin Kyoung, Kyonghwa Song, Sangbaek Park, Taewon Lim, Jongkook Lee, Hyunmin Kang

**Affiliations:** Central Advanced Research and Engineering Institute, Hyundai Motor Company, 37, Cheoldobangmulgwan-ro, Uiwang-si, Gyeonggi-do 16082 Korea

**Keywords:** Nanofluid, Molecular dynamics simulation, Graphene sheet, Functional group, Dispersion

## Abstract

Nanofluids with enhanced thermal properties are candidates for thermal management in automotive systems, with scope for improving energy efficiency. In particular, many studies have reported on dispersions of nanoparticles with long-term stability in the base fluid, with qualitative evaluations of the dispersion stability via either the naked eye or optical instruments. Additives such as surfactants can be used to enhance the dispersion of nanoparticles; however, this may diminish their intrinsic thermal properties. Here, we describe molecular dynamics simulations of nanofluids containing graphene sheets dispersed in ethylene glycol and water. We go on to suggest a quantitative evaluation method for the degree of dispersion, based on the ratio of the total number of nanoparticles to the number of clustered nanoparticles. Moreover, we investigate the effects of functional groups on the surface of graphene, which are expected to improve the dispersion without requiring additives such as surfactants due to steric hindrance and chemical affinity for the surrounding fluid. We find that, for pure graphene, the degree of dispersion decreased as the quantity of graphene sheets increased, which is attributed to an increased probability of aggregation at higher loadings; however, the presence of functional groups inhibited the graphene sheets from forming aggregates.

## Background

Thermal management is a crucial factor in heat transfer equipment, and determines the energy efficiency. In automotive systems, working fluids such as water, ethylene glycol (EG), propylene glycol, engine oil, mineral oil, kerosene oil, and silicon oil are indispensable for effective thermal management; however, enhancements in the thermal conductivity of these fluids are desirable for improved energy efficiency [1]. Dispersing a small quantity of millimeter- or micrometer-sized solid particles in fluids is not practical due to problems associated with sedimentation, erosion, and fouling of flow passageways.

The concept of a “nanofluid” was first reported by Choi [2] at Argonne National Laboratory, and has attracted considerable interest over the past two decades because of the significant enhancements of the heat [3] and mass transfer [4] coefficients, as well as improved wetting and spreading [5]. Dispersions of nanometer-sized solid particles in a base fluid can achieve large enhancements in the thermal conductivity compared with the base fluid alone. For example, Choi et al. [6] reported a 40 % enhancement in the thermal conductivity of ethylene glycol via the addition of 0.3 vol.% Cu particles, as well as a 150 % enhancement of the thermal conductivity of synthetic oil using a 1 vol.% dispersion of carbon nanotubes.

Various nanomaterials have been used as additives in nanofluids, including metallic nanoparticles [6, 7], metallic oxide particles [8, 9], and TiO_2_ nanotubes [5]. Carbon allotropes such as fullerenes, carbon nanotubes, graphene, and graphite also represent candidate materials for additives in nanofluids due to their excellent physical properties. Carbon nanotubes [10–12], exfoliated graphite [13], and carbon nanofibers [14] are particularly promising materials. Graphene is a flat monolayer of *sp*^2^-hybridized carbon atoms that form a honeycomb lattice, and has attracted much interest due to its exceptional physical and chemical properties [15]. It exhibits unusual thermal behavior, with a long-range ballistic transport at room temperature [16]. Baladin et al. [17] reported that graphene exhibits significantly higher thermal conductivity than carbon nanotubes, which gives graphene potential for use in heat management applications with high-power electronics devices. Baladin et al. also proposed a lattice thermal conductivity model of graphene using the framework of the Klemens approximation [18]. They found that the phonon mean free path was 775 nm at room temperature, and that the phonon thermal conductivity of single-layer graphene was in the range 2000–5000 W/mK, and depended on the defect concentration, roughness of the edges, and the flake width [19–23]. Although the potential applications of nanofluids are promising, the long-term stability of nanofluids is a problem [24].

Long-term stability can be described as the duration of a dispersion of nanoparticles in a base fluid, and is a key in determining the physical properties of nanofluids [24]. Nanoparticles in nanofluids exhibit Brownian motion, and (in the absence of a net flow) are characterized by the buoyancy force and thermal agitation. However, agglomeration of nanoparticles results in microparticles settling at the bottom due to gravity, and eventually leads to a loss of function of the nanofluid. There are several methods to improve the dispersion of nanoparticles in a base fluid. The use of carbon-based materials, as discussed above, typically requires a surfactant to wrap each nanoparticle and inhibit aggregation due to van der Waals forces [25]. However, these (at least partially) cover the surface of the nanoparticles, which influences the physical properties; therefore, it is desirable to avoid the use of surfactants.

Several experimental methods have been used to evaluate the dispersion of particles in a base fluid, including observations with the naked eye and the use of optical instruments including ultraviolet–visible–infrared (UV–vis-IR) spectroscopy, zeta potential, and Turbiscan [9]. These methods, however, do not allow quantitative evaluations of the degree of dispersion, which is desirable for reliably fabricating well-dispersed nanofluids.

Here, we describe molecular dynamics (MD) simulations of nanofluids formed of a 1:1 mix of water and EG containing various loadings of graphene. The simulated data allow us to evaluate quantitatively the degree of dispersion of graphene sheets via the inter-centroid distance between sheets. Furthermore, we investigate improvements in the dispersion via modifications of the graphene surface with various functional groups. We found that the degree of dispersion decreased with the increasing loading of graphene sheets because of the increased probability of aggregation at higher loadings; however, functionalized graphene sheets did not exhibit aggregation.

## Methods

The base fluid investigated was a common coolant used in automotive systems, i.e., a mixture of ethylene glycol and water, with a mass ratio of 1:1. Simulations were carried out with various numbers of graphene sheets to vary the mass fraction of graphene. We used the condensed-phase optimized molecular potentials for atomistic simulation studies (COMPASS) force field potential in the software package Materials Studio (ver. 8.0; Accelrys Software Inc., San Diego, CA, USA).

**Fig. 1:**
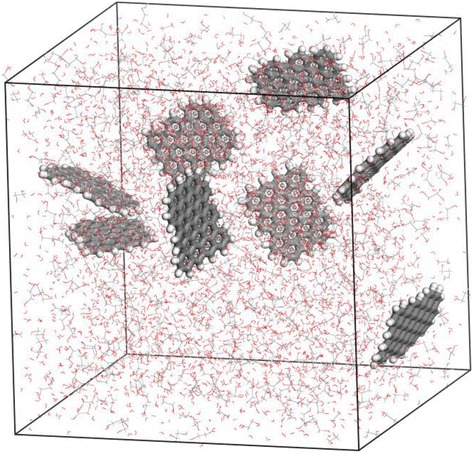
A snapshot of a molecular dynamics simulation of a 5.3 wt.% dispersion of graphene in a 1:1 mixture of water and ethylene glycol (EG)

Figure 1 shows a snapshot of an MD simulation of a nanofluid-containing graphene sheets. The simulation domain consisted of a cubic cell, and the length was varied in the range 5.67–6.18 nm, giving a volume of 181.92–235.93 nm^3^. The dimensions of the cell were determined based on the desired graphene loading. The density was constant at 1 g/cm^3^ (which is consistent with both EG and water). Graphene sheets without functional groups consisted of hexagonal lattices of 61 *sp*^2^-bonded carbon atoms, with 21 hydrogen atoms at the edges. Alcohol and amide (amine) functional groups, having –OH and –N—chemically modified from hydroxyl, carboxylic, and epoxide groups essentially produced during hummer’s method, were attached to the graphene sheets in the simulations of the position of these functional groups in this study were arbitrary determined with considering their degree of freedom for the motion preventing interruption from other functional groups.

The graphene sheets were randomly dispersed to create graphene loadings in the base fluid in the range 1.04–25.4 wt.%. The simulations were performed via an *NVT* ensemble method with 1 fs of time step, where *N* is the number of atoms, *V* is volume, and *T* = 298 K is the temperature, which was set using the Nosé–Hoover–Lagevin (NHL) thermostat method. The simulations were performed for 1 ns, which was found to be sufficient to stabilize the nanofluid. Stabilized total energy of the system for simulation represents whether it reaches in equilibrium state or not. We judged it not only by energy of the system in equilibrium state, but also by the stabilization of degree of dispersion obtained from calculation at each frame.

## Results and Discussion

### Quantitative Evaluation of the Degree of Dispersion

The dispersibility of nanoparticles in nanofluids can be investigated via observations with the naked eye, via differences in transmittance between dispersed and aggregated nanoparticles, or via UV–vis-IR spectroscopy or optical analysis using a Turbiscan. However, it is difficult to compare these results directly, as the degree of dispersion is not quantified using these methods.

Here, we suggest a quantitative evaluation method for the dispersion of nanoparticles based on the ratio of the number of active suspended particles to the total number of particles. Positional data of graphene sheets were obtained from the MD simulations at each time step, and the centroids of the graphene sheets were used to calculate the separation between sheets.

**Fig. 2:**
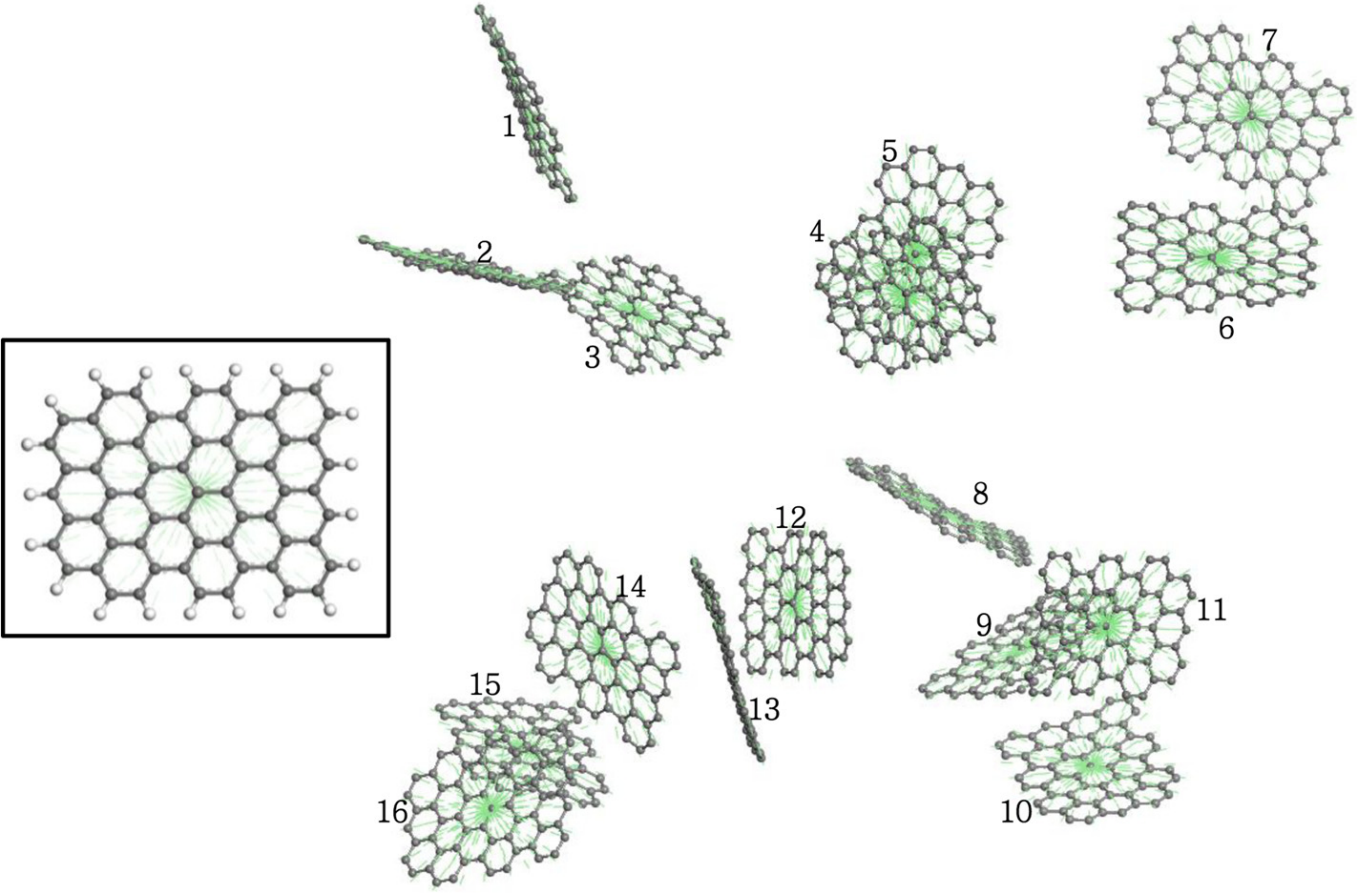
The numbering of centroids of graphene sheets used to calculate the degree of dispersion in a nanofluid. The *inset* shows where the centroid occurs in the graphene sheets. If the separation between graphene sheets becomes smaller than 3.5 times the carbon–carbon Van der Waals separation, the graphene sheets are considered aggregated

The centroids were numbered to prevent double counting, as shown in Fig. 2. We need a factor to decide whether the graphene is dispersed or aggregated, so we made use of the interaction distance between the graphene sheets in order to define the degree of dispersion of graphene. Carbon-based materials tend to be bundled each other due to their strong van der Waals interaction, so the farthest distance limit is applied to judge and calculate the degree of dispersion. When the separation between two graphene sheets was less than 3.5 times the conventional cut-off distance *σ* between non-bonded carbon molecules (determined using the Lennard–Jones potential), they were regarded as agglomerated. The degree of dispersion can be quantified as a number in the range 0–1, i.e.,

1 $$ f=\frac{G_{\mathrm{act}}}{N_{\mathrm{total}}}, $$

where *N*_total_ is the total number of graphene sheets in the base fluid. The number of active graphene sheets is determined by subtracting the number of graphene sheets that have agglomerated to form clusters from the total number of graphene sheets, i.e.,

2 $$ {G}_{\mathrm{act}}={N}_{\mathrm{total}}-{\displaystyle \sum_{i,j}}\left({m}_i-1\right)\cdot {n}_j, $$

where *n*_*j*_ is the number of clusters consisting of *m*_*i*_-aggregated graphene sheets. For example, if there are 100 graphene sheets (*N*_total_) in a given volume where the number of clusters (*nj*) consisting of two, three, and four graphene sheets (*mi*) are two, one and two, respectively, the equation fo *G*_act_ would be *G*_act_=100-[(2-1)x2+(3-1)x1+(4-1)x2]=90. Therefore, the degree of dispersion would be *G*_act_/*N*_total_=90/100=0.9.

### The Effect of Functional Groups

**Fig. 3:**
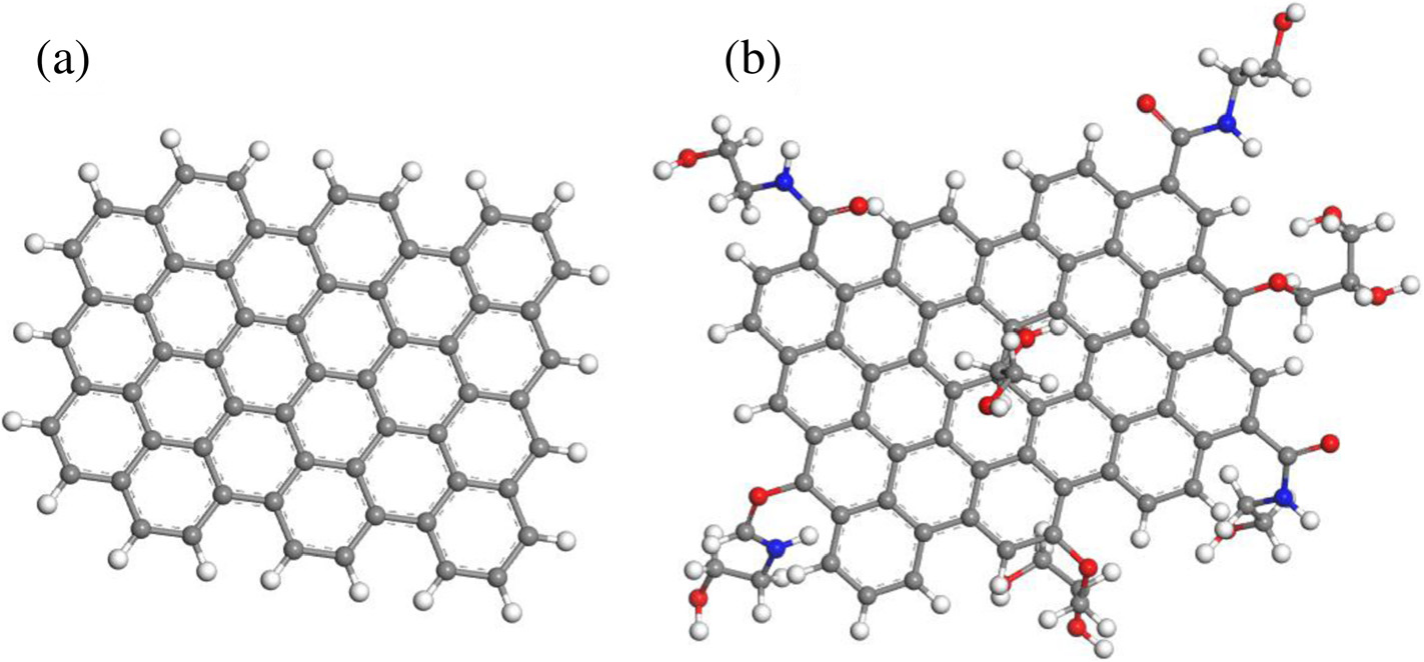
**a** A graphene sheet without, and **b** with functional groups, such as alcohol and amide (amine) having –OH and –N—chemically modified from hydroxyl, carboxylic, and epoxide groups essentially produced during hummer’s method [31]

Figure 3a shows the graphene sheets used in the MD simulations, which consisted of a two-dimensional and hexagonal lattice, with hydrogen atoms bonded at the edges. Well-dispersed nanoparticles in a base fluid can be used to create a nanofluid with enhanced thermal properties, and for this reason, it is desirable to achieve a large degree of dispersion. Graphene tends to aggregate due to strong Van der Waals interactions, and hence external steric hindrance can be used to inhibit aggregation of graphene via chemical additives such as surfactants; however, this may adversely affect the thermal properties by wrapping the individual graphene sheets, creating a significant thermal boundary resistance at each graphene sheet. Many experimental and theoretical studies have reported on the thermal boundary resistance, or Kapitza resistance [26], at the interface between graphene and the surrounding material. This is consistent with the lower-than-expected thermal conductivities that have been reported for many nanocomposites [27, 28].

Chemical exfoliation is a promising method to form graphene sheets from graphite. During this process, various types of oxide functional groups are formed on the surface of graphene sheets, which can influence the dispersion stability in a base fluid. These functional groups can induce hydrogen bonding, stabilizing the dispersion, and providing active sites that enable the formation of heterogeneous structural composites. We investigated three functional groups at the surface of graphene sheets: alcohol and amide (amine) groups, as shown in Fig. 3b, and examined the change in the degree of dispersion via comparison with graphene sheets without functional groups.

**Fig. 4:**
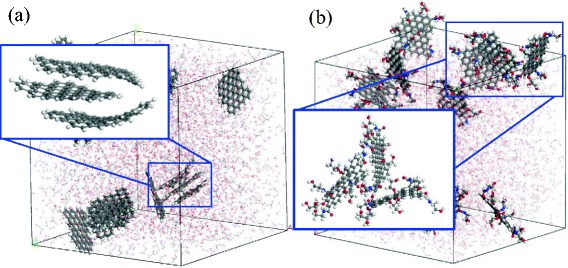
Snapshots of molecular dynamics (MD) simulations for nanofluids containing **a** 10.0 wt.% graphene and **b** 12.4 wt.% functionalized graphene. The enlarged images show that **a** graphene sheets tended to stack to form aggregates, but that **b** the functionalized graphene did not stack

Figure 4a, b shows snapshots of MD simulations of nanofluids containing graphene sheets without and with functional groups, respectively. Without functional groups, the graphene sheets formed agglomerates, whereby individual sheets stacked in the direction normal to the plane of the graphene sheet (see Fig. 4a); however, with the functional groups, the steric hindrance inhibited this stacking behavior (see Fig. 4b).

**Fig. 5:**
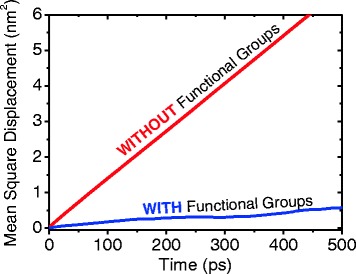
Comparison of MSD between graphene sheet with and without functional groups, at 1.4 and 2.5 wt.%, respectively. The slope of MSD without functional groups is much steeper than that of with. We concluded that this result came from affinity of functional groups to surrounding fluids

On the other hand, we compared a value of the mean square displacement (MSD), or mean square fluctuation between graphene sheets with and without functional groups. In statistical mechanics, it demonstrates the measure of the spatial extent of random motion measuring the portion of the system “explored” by the random walker, and is defined as

3 $$ \mathrm{Mean}\ \mathrm{square}\ \mathrm{displacement} = <{\left(x-{x}_0\right)}^2 > =\kern0.5em \frac{1}{T}{\displaystyle {\sum}_{t=1}^T{\left(x(t)-{x}_0\right)}^2} $$

where *T* is the time for average, and *x*_0_ is the reference position of the particle. Typically, this reference position will be the time-averaged position of the same particle. Figure 5 shows the comparison of MSD between graphene sheet with and without functional groups. The slope of MSD “without” functional groups is much steeper than that of “with”. We concluded that this result came from affinity of functional groups to surrounding fluids.

### The Effect of Graphene Loading

**Fig. 6:**
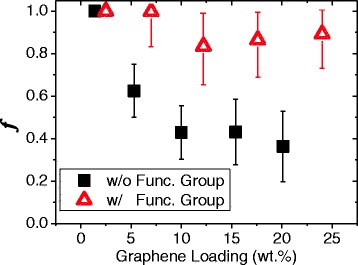
The dispersion ratio *f* as a function of the graphene loading. Without functional groups, the degree of dispersion decreased with increasing graphene loading. With functional groups, however, the degree of dispersion was almost independent of the graphene loading

We investigated the degree of dispersion of nanofluid formed of graphene sheets with and without functional groups at graphene loadings in the range 1–25 wt.%. Figure 6 shows the dependence of the degree of dispersion on the graphene loading with and without functionalization. Without the functional groups, the degree of dispersion decreased nonlinearly as the graphene loading increased, reducing drastically up to 10 wt.%. As the graphene loading increased, agglomeration became more likely. However, as the number of aggregated particles increases, so does the separation between them, and eventually a stable state was reached.

The degree of dispersion of the functionalized graphene sheets exhibited a much weaker dependence on the graphene loading (see the red triangles in Fig. 6). There was little reduction in the degree of dispersion at 12.2 wt.% of graphene loading, and the graphene sheets were in a well-dispersed state. The edges of the graphene sheets contain several functional groups that can be influenced by the surrounding materials. For example, graphene oxide forms a stable dispersion in distilled water, but aggregates in EG. The base fluid used here was a 1:1 (by mass) mixture of water and EG. Functional groups at the edges of the graphene sheets have been suggested to form stable dispersions in water and EG [29]. Furthermore, steric hindrance at the surface of the graphene sheets inhibits staking. Therefore, functional groups at the surface and edges of the graphene sheets are important in maintaining a stable dispersion by inhibiting agglomeration. On the other hand, many studies have reported that the size of graphene in the range from 20 to 50 nm can maximize the nanosize effect which resulted in floating the particle in the fluids [30]. However, it practically takes quite long time to run MD simulation in this range of particle sizes. Therefore, we just used one size of graphene sheet and more focused on the effect of functional groups rather than investigating the effect of the size on the dispersion.

## Conclusions

The concept of a nanofluid was first suggested in 1995, and nanofluids are promising candidates for thermal management in automotive systems. We carried out MD simulations of nanofluids formed of graphene sheets dispersed in a 1:1 mixture of EG and water. In this study, we have suggested an evaluation method to quantify the degree of dispersion of nanoparticles in a base fluid based on the ratio of the total number of nanoparticles to the number of nanoparticles that exist in clusters.

Furthermore, we clearly quantify that the functional groups at the surface of graphene sheets improved the degree of dispersion due to steric hindrance and chemical affinity for the surrounding fluid, without requiring additives. With non-functionalized graphene sheets, the degree of dispersion decreased as the graphene loading increased because of an increased probability of aggregation, whereas the functionalized graphene sheets did not tend to aggregate, even at high loadings.

## References

[1] Sundar LS, Singh MK, Ramana EV, Ramana V, Singh B, Grácio J, Sousa ACM (2014). Enhanced thermal conductivity and viscosity of nanodiamond-nickel nanocomposite nanofluids. Sci Rep.

[2] Choi SUS, Eastman JA (1995). Enhancing thermal conductivity of fluids with nanoparticles. 1995 ASME Int. Mech. Eng. Congr. Expo 231:99–105

[3] Xie H, Wang J, Xi T, Liu Y, Ai F, Wu Q (2002). Thermal conductivity enhancement of suspensions containing nanosized alumina particles. J Appl Phys.

[4] Krishnamurthy S, Bhattacharya P, Phelan PE, Prasher RS (2006). Enhanced mass transport in nanofluids. Nano Lett.

[5] Wasan DT, Nikolov AD (2003). Spreading of nanofluids on solids. Nature.

[6] Eastman JA, Choi SUS, Li S, Yu W, Thompson LJ (2001). Anomalously increased effective thermal conductivities of ethylene glycol-based nanofluids containing copper nanoparticles. Appl Phys Lett.

[7] Warrier P, Teja A (2011). Effect of particle size on the thermal conductivity of nanofluids containing metallic nanoparticles. Nanoscale Res Lett.

[8] Kim SH, Choi SR, Kim D (2007). Thermal conductivity of metal-oxide nanofluids: particle size dependence and effect of laser irradiation. J Heat Transfer.

[9] Lee GJ, Kim CK, Lee MK, Rhee CK, Kim S, Kim C (2012). Thermal conductivity enhancement of ZnO nanofluid using a one-step physical method. Thermochim Acta.

[10] Jyothirmayee Aravind SS, Ramaprabhu S (2012). Graphene wrapped multiwalled carbon nanotubes dispersed nanofluids for heat transfer applications. J Appl Phys.

[11] Harish S, Ishikawa K, Einarsson E, Aikawa S, Chiash S, Shiomi J, Maruyama S (2012). Enhanced thermal conductivity of ethylene glycol with single-walled carbon nanotube inclusions. Int J Heat Mass Transf.

[12] Liu M-S, Lin MC-C, Huang I-T, Wang C-C (2005). Enhancement of thermal conductivity with carbon nanotube for nanofluids. Int Commun Heat Mass Transf.

[13] Yu W, Xie H, Bao D (2010). Enhanced thermal conductivities of nanofluids containing graphene oxide nanosheets. Nanotechnology.

[14] Lee KJ, Yoon S-H, Jang J (2007). Carbon nanofibers: a novel nanofiller for nanofluid applications. Small.

[15] Geim AK, Novoselov KS (2007). The rise of graphene. Nat Mater.

[16] Berger C, Song Z, Li X, Wu X, Brown N, Naud C, Mayou D, Li T, Hass J, Marchenkov AN, Conrad EH, First PN, Heer WA (2006). Electronic confinement and coherence in patterned epitaxial graphene. Science.

[17] Balandin AA, Ghosh S, Bao W, Calizo I, Teweldebrhan D, Miao F, Lau CN (2008). Superior thermal conductivity of single-layer graphene. Nano Lett.

[18] Nika DL, Ghosh S, Pokatilov EP, Balandin AA (2009). Lattice thermal conductivity of graphene flakes: comparison with bulk graphite. Appl Phys Lett.

[19] Nika DL, Pokatilov EP, Askerov AS, Balandin AA (2009). Phonon thermal conduction in graphene: role of Umklapp and edge roughness scattering. Phys Rev B..

[20] Ghosh S, Nika DL, Pokatilov EP, Balandin AA (2009). Heat conduction in graphene: experimental study and theoretical interpretation. New J Phys..

[21] Guo Z, Zhang D, Gong X-G (2009). Thermal conductivity of graphene nanoribbons. Appl Phys Lett.

[22] Hu J, Ruan X, Chen YP (2009). Thermal conductivity and thermal rectification in graphene nanoribbons: a molecular dynamics study. Nano Lett.

[23] Konatham D, Striolo A (2009). Thermal boundary resistance at the graphene-oil interface. Appl Phys Lett.

[24] Yu W, Xie H, Li Y, Chen L, Wang Q (2011). Experimental investigation on the thermal transport properties of ethylene glycol based nanofluids containing low volume concentration diamond nanoparticles. Colloids Surfaces A Physicochem Eng Asp.

[25] Barkoula N-M, Alcock B, Cabrera NO, Peijs T (2008). Fatigue properties of highly oriented polypropylene tapes and all-polypropylene composites. Polym Polym Compos.

[26] Swartz E, Pohl R (1989). Thermal boundary resistance. Rev Mod Phys.

[27] Duong HM, Yamamoto N, Bui K, Papavassiliou DV, Maruyama S, Wardle BL (2010). Morphology effects on nonisotropic thermal conduction of aligned single-walled and multi-walled carbon nanotubes in polymer nanocomposites. J Phys Chem C.

[28] Duong HM, Yamamoto N, Papavassiliou DV, Maruyama S, Wardle BL (2009). Inter-carbon nanotube contact in thermal transport of controlled-morphology polymer nanocomposites. Nanotechnology.

[29] Park M, Lee T, Kim B-S (2013). Covalent functionalization based heteroatom doped graphene nanosheet as a metal-free electrocatalyst for oxygen reduction reaction. Nanoscale.

[30] Kim HJ. (2015) Experimental investigation on the flow and convective heat transfer coefficient of nanofluids in turbulent region. PhD Thesis: Korea Aerospace University.

[31] Hummers WS, Offeman RE (1958). Preparation of graphitic oxide. J Am Chem Soc.

